# Comparative Transcriptomic Analysis of Subcutaneous Adipose Tissue from Local Pig Breeds

**DOI:** 10.3390/genes11040422

**Published:** 2020-04-15

**Authors:** André Albuquerque, Cristina Óvilo, Yolanda Núñez, Rita Benítez, Adrián López-Garcia, Fabián García, Maria do Rosário Félix, Marta Laranjo, Rui Charneca, José Manuel Martins

**Affiliations:** 1 MED-Mediterranean Institute for Agriculture, Environment and Development, Instituto de Investigação e Formação Avançada & Universidade de Évora, Pólo da Mitra, Ap. 94, 7006-554 Évora, Portugal; mlaranjo@uevora.pt; 2 Departamento de Mejora Genética Animal, Instituto Nacional de Investigación y Tecnología Agraria y Alimentaria (INIA), 28040 Madrid, Spain; ovilo@inia.es (C.Ó.); nunez.yolanda@inia.es (Y.N.); rmbenitez@inia.es (R.B.); adrian.lopez@inia.es (A.L.-G.); fabian.garcia@inia.es (F.G.); 3 MED & Departamento de Fitotecnia, Escola de Ciências e Tecnologia, Universidade de Évora, Pólo da Mitra, Ap. 94, 7006-554 Évora, Portugal; mrff@uevora.pt; 4 MED & Departamento de Medicina Veterinária, Escola de Ciências e Tecnologia, Universidade de Évora, Pólo da Mitra, Ap. 94, 7006-554 Évora, Portugal; rmcc@uevora.pt; 5 MED & Departamento de Zootecnia, Escola de Ciências e Tecnologia, Universidade de Évora, Pólo da Mitra, Ap. 94, 7006-554 Évora, Portugal

**Keywords:** Alentejano pig, Bísaro pig, RNA-seq, differentially expressed genes (DEGs), Dorsal subcutaneous fat, transcriptome

## Abstract

When compared to modern lean-type breeds, Portuguese local Alentejano (AL) and Bísaro (BI) pig breeds present a high potential for subcutaneous and intramuscular fat (IMF) deposition which contributes for better meat quality. The aim of this work was to explore the genome function to better understand the underlying physiological mechanisms associated with body fat accretion. Dorsal subcutaneous fat samples were collected at slaughter from adult animals (*n* = 4 for each breed) with ~150 kg body weight. Total RNA was obtained and sequenced for transcriptome analysis using DESeq2. A total of 458 differentially expressed (DE) genes (q-value < 0.05) were identified, with 263 overexpressed in AL and 195 in BI. Key genes involved in *de novo* fatty acid biosynthesis, elongation and desaturation were upregulated in AL such as *ACLY*, *FASN*, *ME1*, *ELOVL6* and *SCD*. A functional enrichment analysis of the DE genes was performed using Ingenuity Pathway Analysis. Cholesterol synthesis is suggested to be higher in AL via SREBF2, SCAP and PPARG, while lipolytic activity may be more active in BI through GH and AMPK signalling. Increased signalling of CD40 together with the predicted activation of INSIG1 and INSIG2 in BI suggests that this breed is more sensitive to insulin whereas the AL is less sensitive like the Iberian breed.

## 1. Introduction

Pork meat represents one of the main sources of protein, fat and iron for humans, accounting for about 30% of meat consumption worldwide. Fresh meat market is essentially dominated by a few genetically selected breeds with notable productive traits and raised under intensive conditions [1]. However, in the last decades, a growing interest for better meat quality has increased the tendency for native breeds to prosper [2,3]. These breeds are generally well adapted to the local environment and subsist in small populations with diversified and accessible food from natural resources. Alongside their inherent genetic value for biodiversity, local breeds are used to produce high quality dry-cured meat products, representing an important role in local economies, culture and landscape as the basics of sustainable local pork chains [2,4].

Alentejano (AL) and Bísaro (BI) are the two main Portuguese pig breeds. AL evolved from the primitive *Sus scrofa mediterraneus*, belongs to the Mediterranean group of breeds [5] and is genetically similar to the Iberian (IB) pig [6]. This breed is commonly raised in the south of Portugal and generally characterized by its light bone structure, black color, short and slim extremities and energetic nature [7]. A medium-sized pig, the AL grows at a low rate (except under the finishing phase “montanheira”) and presents a low prolificacy [8]. On the other hand, its high and early adipogenic activity provides a meat and fat composition that is attractive for both fresh meat market and for processing high-grade sausage and dry cured products [9,10]. Traditionally, the AL pig is raised under extensive conditions in an integrated pastoral system (“montado”) and during the fattening season is fed with acorns from the existing Quercus forests from October to February [5,7]. BI breed belongs to the Celtic group [5], sharing ancestors with highly productive breeds such as Large-White and Landrace. Production of BI is distributed throughout the north of Portugal, from the Tagus River to the northern border with Spain. It is characterized by its docile temperament, presenting a large body with large legs, head and shoulders. BI pig presents a higher prolificacy and productivity than AL, though lower than other genotypes with similar origins but raised under intensive production systems and subjected to genetic improvement programs [11,12,13,14]. On the other hand, the BI pig presents a lower adipogenic trend when compared to AL, but still higher than most modern lean breeds, which leads to a medium fat carcass with an overall good sensorial quality of meat and capability to further process into high-grade meat products [15,16]. Traditionally, BI production is based on small-scale family farms with a low number of sows, fed with domestic food scraps and horticultural by-products [17,18]. Nowadays, the number of sows per farm has increased, while reared by smallholders and in medium-sized farms based on free-ranged systems, most of them allowing access to pasture [19].

The old perception of adipocytes as a mere energy storage tissue is nowadays incomplete due to its extensive autocrine, paracrine and endocrine activities via liberation of various specific cytokines that modulate gene expression and nutrient flow to balance current metabolic needs [20]. Identification of these mechanisms and how they interact can help understand and treat associated metabolic diseases such as obesity and type-2 diabetes. Meanwhile, adipocyte size and number set the overall fatness attribute of a carcass that is essential for the marbling trait preferred by consumers which is described by the presence of an acceptable amount of intramuscular fat (IMF) [21]. Subcutaneous fat, on the other hand, is found beneath the skin in several layers separated by connective tissue, with the breeds overall potential for fat deposition affecting the full development of the most inner layer [21].

Lipid accumulation and further deposition precede a shift in the metabolic balance, favoring lipogenesis and adipogenesis over catabolic pathways such as β-oxidation and is consequence of an excessive caloric intake. The AL, as the Iberian pig and other Mediterranean breeds, feature a genetic predisposition to gain and preserve subcutaneous and IMF, referred as the thrifty genotype [22]. This susceptibility was historically advantageous when animals had to endure seasonal periods of starvation and the excess fat stores developed during periods of abundance were valuable sources of energy for survival [23]. Fatter animals generally present increased levels of circulating leptin and insulin. Leptin is secreted by the adipocytes as an attempt to control appetite and food intake [24], however humans and animals naturally propense to become obese, such as the IB pig, are found to display a pattern of resistance to leptin [25]. In IB pigs, leptin-resistance is at least partially justified by the presence of a fixed functional polymorphism in the leptin receptor gene [26,27] which was recently found as almost fixed in the AL (0.98) and at a much lower frequency in the BI breed (0.26) [6]. Obese animals also secrete more insulin in order to control circulating glucose levels by inducing glucose uptake and storage as glycogen, while triggering lipogenesis and preadipocyte differentiation [28,29]. On the other hand, insulin sensitivity has been previously proposed as a major factor to justify differences in phenotypical traits such as growth and body composition between local and lean modern breeds [30]. A recent study did, in fact, determine that growing IB pigs can develop insulin resistance at an early stage and that insulin is less effective in IB than in Landrace pigs [30]. A similar pattern of diminished insulin sensitivity and possible insulin resistance is therefore possible to develop in AL, particularly when compared to the leaner BI pig. Insulin resistance is characterized by an over secretion of insulin resulting in hyperinsulinemia that induces triglyceride accumulation and lipolysis inhibition in tissues via glucose transporter-4 (GLUT4), mediated by the phosphatidylinositol 3-kinase (PI3K) signaling pathway [29,31]. Furthermore, obese insulin resistant animals secrete more proinflammatory cytokines, which in turn contribute to the development of an obesity-induced chronic inflammation state [32]. These attributes show the potential and justify the continuing application of local pig breeds as biomedical models to study worldwide growing metabolic related disorders such as obesity, type-2 diabetes mellitus and cardiovascular diseases [25].

Genetic potential, along with specific feeding and production strategies, plays a major role on the development and composition of tissues. This work was intended to explore the genome function of AL and BI pig breeds at the level of adipose tissue, to better understand the underlying mechanisms associated with lipid deposition and productive traits of these breeds. This RNA-seq comparative analysis represents the first high-throughput transcriptomic data involving these local breeds and can help explain the metabolic differences that occur in their adipose tissue.

## 2. Materials and Methods

### 2.1. Animals, Experimental Design and Sampling

Purebred male castrated AL and BI pigs (*n* = 4 for each breed) were reared in a traditional free ranged system and individually fed commercial diets ad libitum until slaughter (~150 kg), under identical conditions to minimize non-genetic effects. AL pigs averaged a total of 155.0 days on trial while BI pigs averaged a total of 140.0 days on trial. Dorsal subcutaneous fat samples (DSF) were collected at slaughter as previously described [33], snap frozen in liquid nitrogen and maintained at −80 °C until total RNA extraction.

All animals were raised and slaughtered in conformity with the regulations and ethical guidelines of the Portuguese Animal Nutrition and Welfare Commission (DGAV, Lisbon, Portugal) following the 2010/63/EU Directive. Staff members of the team involved in animal trials were certified for conducting live animal experiments delivered by the Directorate of Animal Protection (DSPA, DGAV, Lisbon, Portugal).

### 2.2. RNA Extraction and Sequencing

Total RNA was isolated from 50–100 mg samples of DSF following Ambion^®^ RiboPure™ Kit (Thermo Fisher Scientific, Waltham, MA, USA) instructions. Total extracted quantity was measured using NanoDropTM 1000 spectrophotometer (Thermo Fisher Scientific, Waltham, MA, USA). Quality control was assessed using Agilent 2100 BioanalyzerTM (Agilent Technologies, Santa Clara, CA, USA) following Agilent RNA 6000 Nano Kit instructions, along with NanoDropTM 1000 260/280 and 260/230 coefficients that were checked for protein and chemical contamination, respectively. RIN values ranged from 6.5~8.5. The obtained total RNA was diluted into a concentration of 100 ng/µL and ~3 µg samples were sent for stranded paired-end mRNA-seq sequencing in Centro Nacional de Análisis Genómico (CNAG-CRG, Barcelona, Spain) on a HiSeq2000 sequence analyzer (Ilumina, Inc., San Diego, CA, USA). The raw data was downloaded from CNAG servers and treated accordingly.

### 2.3. Quality Control, Mapping and Assembly

FastQC program (version 0.11.8) [34] was run to assess the quality of the sequencing Fastq files. Sequence reads were trimmed three consecutive times for Ilumina adapters, poli-A and poli-T tails using Trim Galore (version 0.5.0) [35] while removing resulting low quality nucleotides (Phred Score, Q < 20) and short length reads (<40). The remaining reads were aligned to the reference pig genome version Sscrofa11.1 (Ensembl release 94) using HISAT2 version 2.1.0. Resulting SAM files were then converted to BAM with Samtools-1.9 [36] and HTSeq-count version 0.11.1 [37] was used to count and merge reads based on overlapping paired-end reads.

### 2.4. Differential Expression Analysis

Previously generated Gcount files were run with the R package DESeq2 [38]. In this tool, gene expression levels are obtained through count of total exon reads for the statistical analysis. DESeq2 sets up normalized counts that were filtered by the rule of minimum 50 reads per group. Genes were considered as DE when presenting a false discovery rate (FDR) or adjusted *p*-value lower than 0.05. The data discussed in this publication have been deposited in NCBI’s Gene Expression Omnibus [39] and are accessible through GEO Series accession number GSE145956 (https://www.ncbi.nlm.nih.gov/geo/query/acc.cgi?acc=GSE145956).

### 2.5. Functional Enrichment Analysis

To explore causal relationships associated with the resulting DE genes, predict downstream effects, identify new targets and better interpret their biological meaning within the adipose tissue context, a functional enrichment analysis was performed using Ingenuity Pathways Analysis software (IPA; QIAGEN). The list of candidate DE genes (q < 0.05) and their respective log2 FC ratio was uploaded into the application and then converged with IPA’s library (Ingenuity Pathway Knowledge Base) to identify biologically relevant information such as overrepresented pathways and functions, networks and regulators [40].

### 2.6. Real Time Quantitative PCR and Statistical Analysis

Previously extracted total RNA was reverse transcribed in 20 µL reactions using Maxima^®^ First Strand cDNA Synthesis Kit for RT-qPCR (Thermo Scientific, Waltham, MA, USA) following manufacturer’s instructions. In order to validate the data generated by RNA sequencing, Real Time-qPCR was performed for a set of 9 candidate genes including *ACACA*, *ACLY*, *ADIPOQ*, *ELOVL6*, *FASN*, *LEP*, *ME1*, *PCK1* and *SCD* (Supplementary Table S1). Standard PCRs were executed to check amplicon sizes.

Quantification reactions containing 12.5 μL of NZY qPCR Green Master Mix (2×) (NZYtech, Lisbon, Portugal), 0.3 μM of each respective sense primer and 12.5 ng of cDNA per sample were prepared in 96-well plates and run in a LineGene9600 Plus system (BIOER, Hangzhou, China). PCR program comprised an initial hold 10 min denaturation stage at 95 °C, followed by a 40 cycles amplification step of 15 s denaturation at 95 °C and 50 s at the respective primer pair annealing temperature (Supplementary Table S1). A melting stage to test PCR specificity was also added at the end involving a single cycle at 95 °C (15 s) followed by 60 °C (60 s), and a ramp-up 0.2 °C/s to 95 °C for 15 s with acquired fluorescence. Single peaks in the dissociation curves confirmed the specific amplification of the genes. A no-template control was run with every assay, and target samples were performed in triplicate as technical replicates. Ct values were regressed on the log of template cDNA concentration. For each gene, PCR efficiency was estimated by standard curve calculation [41] using five points of cDNA serial dilutions (1:4; 1:8; 1:16; 1:32; 1:64). *ACTB*, *RPL19* and *TOP2B* were selected as endogenous genes for normalization of target genes and their stability was assessed using the Genorm software (0.271 < M < 0.363) [42].

An Independent Samples t-test was performed on the dataset using IBM SPSS Statistics software (IBM SPSS Statistics for Windows, Version 24.0. Armonk, NY: IBM Corp) with significance defined as *p* < 0.05. Equal variances were tested with Levene’s Test for Equality of Variances. Significance levels lower than 0.05 were not considered as equal variances and another Independent Samples Test was performed assuming no equal variances. Equal variances were not assumed for *ACLY* (F = 12.5; *p* = 0.01) and *ME1* (F = 32.3; *p* = 0.001). Pearson correlation coefficients and associated *p*-values were estimated.

To measure the level of agreement between RNA-seq and Real time qPCR results, the concordance correlation coefficient (CCC) [43] was estimated using the Log2FC values per candidate gene.

## 3. Results and Discussion

### 3.1. Alentejano and Bísaro: Local Pig Breeds with Recognizable Meat Quality Traits

The animals tested in this study, purebred AL and BI pigs differ phenotypically, genetically and regarding their respective traditional production systems. These breeds represent the two most important local pig breeds produced in Portugal, and there is a renewed interest in their resulting crosses, the Ribatejano (RI) pig [11]. In a previous study [33], a comparison of productive and meat quality traits was analysed in the AL, BI and RI pigs, with BI presenting significantly better carcass traits than AL and intermediate values for the crossed pigs. From this study, four randomly selected individuals from AL and BI breeds were chosen for transcriptome analysis by RNA-seq. Currently, many RNA-seq experiments are performed at a low replication level and several publications suggest that a minimum of 2–3 replicates can be considered [44,45,46]. Selected AL pigs averaged a total of 155.0 days on trial with an average daily gain of 571.6 g/d while BI pigs averaged a total of 140.0 days on trial and an average daily gain of 619.4 g/d. On the other hand, when compared to BI, AL pigs presented significantly higher fat cuts proportions (32.0 vs. 25.4%, *p* < 0.05), average backfat thickness (78.6 vs. 45.1 mm, *p* < 0.01) and fat depth (69.6 vs. 36.5 mm, *p* < 0.01). BI pigs on the other hand presented higher primal cuts proportions (33.2 vs. 26.1%, *p* < 0.01), bone cuts proportion (13.8 vs. 11.8%, *p* < 0.05) and lean-to-fat cuts ratio (1.89 vs. 1.41, *p* < 0.05).

### 3.2. Mapping and Annotation

The average number of obtained sequenced reads was 45 million per sample. Length of reads was consistently 76 bp with the average associated quality score roughly close to 40. GC content ranged from 49 to 56%. Resulting trimmed paired-end reads were mapped to the reference genome Sscrofa11.1 with a consistent alignment rate of 97% using HISAT2 over all samples, which is higher than most previous pig transcriptome studies [47,48,49,50,51,52] and is probably due to an improved annotated reference genome.

### 3.3. Gene Expression Analysis: DESeq2

Previously obtained normalized counts were used to predict and establish the total number of DEG’s between breeds. Over 10.7 K genes were detected across all samples with 458 found to have significantly different expression values between breeds (q < 0.05). A total of 263 genes were found overexpressed in AL and 195 in BI with 47 (20%) of total DEG’s still labelled as novel genes with hardly any information available on most databases. A full detailed list of the total DEG’s can be found in Supplementary Table S2. The novel gene coding for the baculoviral inhibitor of apoptosis protein repeat containing 7 (*BIRC7*) was the most overexpressed in the AL breed (log2 FC = 4.86, q < 0.01) while Taste receptor type 2 member 39 (*TAS2R39*) was the most overexpressed in BI (log2 FC = −4.61, q < 0.05). In humans, *TAS2R39* plays a role in the perception of bitterness and is linked with the G protein associated with taste and the gustatory system [53]. In pigs, taste perception can influence feeding patterns which can consequently determine production traits and *TAS2R39* in particular has been previously associated with increased lipid deposition [54]. A recent study that investigated the allele frequency of known polymorphisms with associated meat quality traits in European local pig breeds [6], including AL and BI, found that the missense mutation p.Leu37Ser in *TAS2R39* associated with higher backfat deposition is practically absent in AL (0.01), while in BI is present in 13% of individuals. This contradicts phenotypic data and suggests that regulation of lipid content may occur at different levels and differ in pathways from AL pigs.

**Figure 1:**
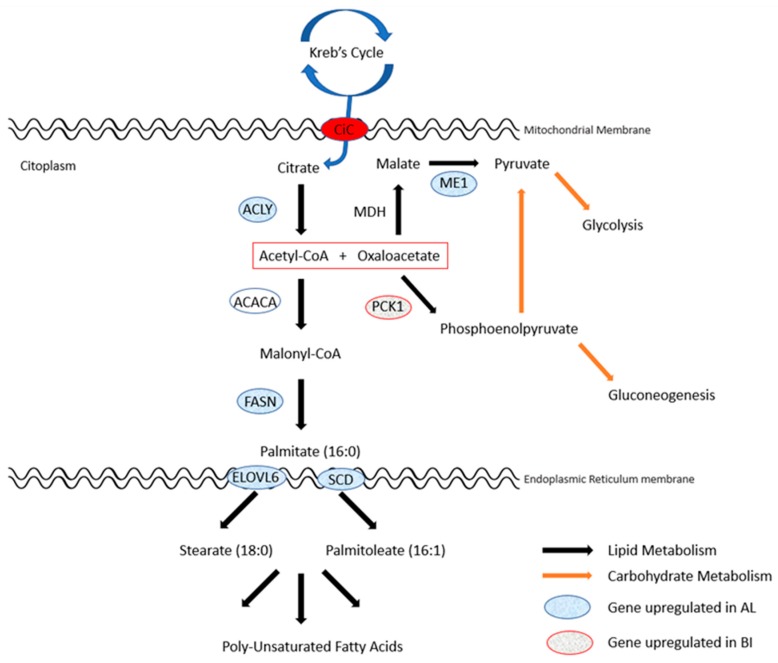
Linking lipid biosynthesis with carbohydrate metabolism: direction of upregulated genes in the Portuguese local pig breeds.

Overall, and as expected, genes encoding for enzymes or transcription factors involved in lipid synthesis were found overexpressed in AL. Furthermore, we confirmed that, in this breed, several major genes involved in the cascade of the de novo lipid synthesis, elongation for very long fatty acids and desaturation, were upregulated (Figure 1).

ATP citrate lyase (*ACLY*) was found significantly overexpressed in AL when compared to BI (log2 FC = 1.853, q < 0.01). ACLY is the primary enzyme responsible for the synthesis of cytosolic acetyl-CoA in many tissues. It catalyses the reaction where the citrate produced through Krebs Cycle, freely transported to the cytoplasm by the Citrate Carrier (CiC), is converted into acetyl-CoA and oxaloacetate [55,56], along with the hydrolysis of ATP, linking the carbohydrate to the lipid metabolisms. Acetyl-CoA is the main non-lipid precursor, with NADPH, for the synthesis of cholesterol and/or palmitic acid through *de novo* synthesis [57].

The first step to synthesize new fatty acids comprises the carboxylation of acetyl-CoA into malonyl-CoA [57], a reaction catalysed by the enzyme acetyl CoA carboxylase (ACACA) who’s gene was found in our data to be non-statistically different between breeds though a numerical difference was noted (log2 FC = 0.854, q = 0.116). That difference was then confirmed and boosted to trend by qPCR (log2 FC = 1.055, *p* = 0.077).

Long carbon chain FAs are then assembled in multiple repeated four-step cycles comprehending: condensation, reduction, dehydration, and reduction. In each cycle two carbon atoms are added to the acyl chain, which is the substrate for the next cycle with an activated malonyl CoA group [58]. For the complete synthesis of palmitic acid (16:0) the cycle must be repeated seven consecutive times, a reaction operated by the multifunctional enzyme complex fatty acid synthase (FASN) that requires much NADPH. In our data, *FASN* was overexpressed in AL (log2 FC = 1.691, q < 0.01) validating the concept of an increased lipid biosynthesis in AL when compared to BI.

Elongation of saturated and/or unsaturated FAs occurs in the cytoplasmic face of the endoplasmic reticulum membrane where the elongation of very long chain fatty acids protein 6 (ELOVL6) plays a pivotal role by adding two-carbon atoms from a malonyl CoA donor [59]. *ELOVL6* was found also significantly overexpressed in AL (Log2 FC = 1.236; q < 0.01) suggesting a higher production of long chain FAs, particularly stearic (18:0) and oleic (18:1) acids.

The acyl CoA desaturation also occurs at the endoplasmic reticulum. Stearoyl CoA desaturase (SCD), together with NADH-cytochrome b_5_ reductase and cytochrome b_5_, work as a membrane-bound complex to catalyse the reaction that introduces a double bond between C9 and C10 [60] to mainly create palmitoleic (16:1) and/or oleic acid which are major components of membrane phospholipids, triacylglycerols and cholesterol esters [58,60]. Furthermore, oleic acid is the starting point to assemble a variety of unsaturated FAs by a combination of elongation and desaturation reactions. SCD is the rate-limiting step of the reaction and, in our data, *SCD* gene was also found significantly overexpressed in AL (log2 FC = 2.311, q < 0.026). This suggests a higher synthesis of oleic acid and its derivatives in this genotype, which lead to a higher oleic acid content in meat as found in previous studies [7,9,61].

When citrate is converted to acetyl-CoA and oxaloacetate, the later can be converted to malate through the action of malate dehydrogenase (MDH) and then to pyruvate to enter the glycolytic pathway [62]. The latter reaction is catalysed by malic enzyme 1 (ME1) and is characterized by the reversible oxidative decarboxylation of malate generating much of the NADPH supply required for lipid biosynthesis [63]. In our data, *ME1* gene was also found significantly overexpressed in AL (log2 FC = 2.090, q < 0.01) supporting the idea of an overall increased lipid synthesis in this breed.

Phosphoenolpyruvate carboxykinase 1 (PCK1) is responsible for catalysing the reaction where cytosolic oxaloacetate is converted to the intermediate phosphoenolpyruvate which can participate in gluconeogenesis or glycolysis when converted to pyruvate [57]. Moreover, PCK1 has been found to regulate free fatty acid reesterification and glyceroneogenesis in white adipose tissue [64]. Contrarily to what was found in another study using Iberian when compared to Duroc pigs [52], *PCK1* gene was found significantly overexpressed in BI (log2 FC = −1.959, q < 0.05) which suggest that in BI the path of gluconeogenesis/glyceroneogenesis pathway is favoured. A single nucleotide polymorphism c.A2456C in the *PCK1* gene has been reported to favour higher intramuscular deposition and better meat quality in pigs muscle [65]. The presence of this mutation in AL and BI genotypes was recently studied [51] showing that AL presents the A allele practically fixed (0.97) as do the IB (0.96). However, the C allele is present in BI in approximately 50% of the animals. The C allele causes the integration of leucine instead of methionine at the position 139 of the enzyme, which is associated with higher meat water loss and less favourable fat distribution. PCK1 p.139Met is kinetically more active in the direction of phosphoenolpyruvate synthesis for glyceroneogenesis while the PCK1 p.139Leu (allele A) is more active in the opposite direction, leading to oxaloacetate synthesis [65] which may contribute to the less fatty phenotype of the BI breed contrasting to the higher lipid deposition and lower carcass traits.

Leptin (LEP) is a homeostatic hormone mediator mainly expressed in adipocytes. It is widely recognized for regulating food intake and energy expenditure at the hypothalamic level [66] although the exact working mechanism in the adipose tissue continue unclear. Circulating levels of LEP are consistently higher in fatty animals. LEP targets peripheral tissues favouring FA catabolism over lipogenesis and it has been suggested that leptin can regulate adipose tissue metabolism by autocrine signalling [67]. As in other studies with the Iberian pig breed [52,68,69], genetically similar to AL, compared to the leaner Duroc and Landrace breeds, *LEP* gene was found significantly overexpressed in the AL pig (log2 FC = 1.376, q < 0.05). This is in agreement with its recognized fatter phenotype and, very similar to the IB breed, suggesting the development of leptin resistance [27,52].

The CCAAT/enhancer-binding protein α (*CEBPA*) gene encodes a transcription factor, containing a basic-leucine zipper domain that recognizes the CCAAT sequence in the promoter region of specific target genes that indirectly participates in the regulation of multiple pathways, including glucose and lipid metabolism [70,71]. In humans, several studies have found a lower expression of *CEBPA* in obese groups [72,73] despite being usually associated to adipogenesis, adipose tissue development and lipid accumulation [71,74]. However, in our study, *CEBPA* was found upregulated in the obese AL (log2 FC = 0.974, q < 0.05) supporting the concept of increased lipid synthesis in this breed.

The fatty acid binding protein 4 (*FABP4*) gene encodes for a cytoplasmic protein found in adipocytes that binds long-chain fatty acids and plays an essential role in lipid metabolism and homeostasis. In cattle, FABP4 has been associated with better marbling and fat depth [75] and in pigs is a recognized genetic marker for meat tenderness and IMF content [76]. FABP4 is also known to indirectly influence adipogenesis and insulin responsiveness by adjusting the master regulator peroxisome proliferator-activated receptor gamma (PPARG) [77]. In our data, *FABP4* was found significantly overexpressed in AL (log2 FC = 1.061, q < 0.01), which agrees with the frequently higher expression values found in obese individuals [77]. Higher FABP4 levels are induced by increased levels of insulin or insulin-like growth factor-1 (IGF1) and are frequently associated with the development of insulin resistance [78].

Adipocyte expression of IGF2 has been previously associated with enhanced foetal growth and subcutaneous preadipocyte differentiation via PPARG activation while decreasing fat deposition in visceral preadipocytes [79]. In pigs, *IGF2* is a candidate gene for meat production and carcass traits [80]. An intronic mutation in *IGF2* (g.3072G > A) is strongly related to fat depot and muscle development when the G allele is present [81]. In AL pigs the G allele is fixed while in BI the A allele is residual (0.01) [6]. *IGF2* was found significantly overexpressed in BI (log2 FC = −0.884, *p* < 0.05) despite their leaner phenotype which indicates that fat deposition is regulated at other levels. This result is in agreement with previous studies that found IGF2 overexpression in leaner Duroc when compared to the fatter Iberian pig genotype [52].

Similar to what was found in a recent study [52] that compared the transcriptome of animals with distinct tissue distribution (Iberian vs. Duroc), a set of genes that play an important role in growth and development was, within our dataset, consistently found overexpressed in BI, the breed with the highest lean muscle tissue deposition. Those genes included *IGF2* (log2 FC = −0.884, q < 0.05), *FOS* (log2 FC = −1.087, q < 0.05) and *FOSB* (log2 FC = −2.028, q < 0.01). Moreover, several other genes associated with proliferation of muscle cells and cellular development were consistently found overexpressed in this local breed, including *APOD* (log2 FC = −1.087, q < 0.05), *DUSP1* (log2 FC = −1.539, q < 0.01), *EGR1* (log2 FC = −1.172, q < 0.05), *ELN* (log2 FC = −1.743, q < 0.01), *KLF4* (log2 FC = −1.103, q < 0.05) and *STAT3* (log2 FC = −0.940, q < 0.05). These findings agree with other previous works [49,52,82] and support the idea that growth and development stimuli, particularly in the more productive breeds, can occur and be regulated at a multiple tissue level.

### 3.4. Validation by Real Time qPCR

In order to validate the RNA-seq results, the relative expression of a selected group of DE genes as well as some non-DE genes was assessed by semi-quantitative Real Time PCR within the same sampling universe (Table 1). Overall, RNA-seq results agreed with the results obtained from real time qPCR, despite occasional small significance inconsistencies within some genes probably due to particularities of each method regarding accuracy, sensitivity and specificity.

As found in other studies [49], statistically significant differences generally tend to be higher with the RNA-seq approach when compared to the Real-Time qPCR technology where *p*-values are higher. A concordance correlation coefficient (CCC), usually employed to measure the degree of agreement between two variables in order to evaluate the reproducibility of tested methods, was determined between the RNA-seq and qPCR methods. The obtained value of 0.804 demonstrates a substantial strength-of-agreement between the treatments.

### 3.5. Functional Analysis

IPA software was used to find overrepresented biological functions, pathways and potential upstream regulators within the candidate DEG dataset, where 380 of the total 458 candidate DEG’s were recognized by its database.

Gene Ontology enrichment analysis revealed a total of 500 involved biological functions (*p* < 0.05) within our DEG dataset (Supplementary Table S3), with 17 of these being significantly activated (z-score ≤ −2 or ≥2) in a breed. A total of 5 functions related either to development and growth or cell death were predicted to be activated in BI (z-score ≤ −2) including generation of embryonic cell lines, necrosis and cell hypoplasia. On the other hand, 12 biological functions mostly related to cell-signalling and cellular development were predicted to be activated in AL (z-score ≥ 2), including cell proliferation of hepatoma cell lines, activation of myeloid cells, proliferation of fibroblast cell lines and activation of granulocytes. Other biological functions of interest were found significantly associated to our gene dataset, but no directional prediction was attained. Among them, size of animal, gluconeogenesis, quantity of glycogen, synthesis of lipid, concentration of fatty acids, quantity of white adipose tissue, concentration of triacylglycerols, synthesis of sterols, proliferation of muscle cells, inflammatory response and insulin sensitivity. As reported in a similar study that compared the breed effect on the adipose tissue transcriptome [52], our results suggest that a highly activated inflammatory state, frequently defined as meta-inflammation, may have developed in the adipose tissue due to the large fat mass attained, particularly in AL pigs.

A total of 57 canonical pathways were found associated with our DEG dataset (*p* < 0.05) (Supplementary Table S4). Two of them were found within the scope of a directional prediction (z-score ≤ −2; z-score ≥ 2), namely CD40 signalling (z-score = −2.236) and mouse embryonic stem cell pluripotency (z-score = −2.236). *CD40* encodes for a protein member of the TNF-receptor superfamily which operates as a receptor for immune system cells and participate in the regulation of numerous immune and inflammatory responses [83]. Studies in mice [84,85], have pointed the regulatory role of CD40 in obesity-induced insulin resistance and has associated CD40 deficiency with increased inflammation and decreased insulin sensitivity, pointing its potential in preventing obesity and metabolic disorders. The fact that CD40 signalling is one main canonical pathways found and that is significantly favoured in BI, supports the idea that insulin resistance is mainly occurring in the AL breed, where decreased insulin sensitivity is suggested due to the pro-inflammatory effect of the exacerbated level of adipose tissue deposition when compared to the leaner BI.

The top canonical pathway detected was Growth Hormone (GH) Signalling (*p* = 1.95 × 10^−4^), followed by the HOTAIR regulatory pathway (*p* = 3.80 × 10^−4^), both suggested to be activated in BI (−0.378 and −0.632 of z-score, respectively). GH is widely recognized for its anabolic and lipolytic functions, stimulating muscle and bone growth mediated by IGF1 secretion. In adipocytes, stimulated GH receptors induce lipolysis through oxidation and triacylglycerol breakdown [86]. On the other hand, the homeobox transcript antisense intergenic RNA (HOTAIR) is part of the long noncoding RNAs class that is suggested to regulate the expression of numerous signalling transcripts and is a potential biomarker for several types of cancer [87]. Recent studies have also associated upregulated levels of HOTAIR with increased adipogenesis, through upregulation of *FASN* [88,89], which suggest a different regulatory pathway in BI compared to the AL breed, that presented a higher expression of *FASN*.

Other interesting canonical pathways associated to BI include prolactin (z-score = −0.447, *p* = 0.002) and signalling of a few specific regulators such as AMP-activated protein kinase (AMPK) (z-score = −1.134, *p* = 0.01), IGF1 (z-score = −0.447, *p* = 0.048) and cytokines interleukin-2 (IL-2) (z-score = −1.000, *p* = 0.019) and IL-3 (z-score = −1.000, *p* = 0.019). These pathways suggest a higher lipolytic activity within the BI adipocytes, along with the activation of anti-inflammatory signals that may help control insulin sensitivity. Prolactin plays a major role in lactation and can also regulate the reproductive and immune systems. Metabolically, prolactin has been associated with a reduced ability for glucose and lipid deposition in human adipocytes, leading to higher circulating values of these compounds [90]. Patients with hyperprolactinemia are found with metabolic disorders such as insulin resistance and glucose intolerance [91]. Moreover, prolactin and GH can affect the expression of adiponectin, the adipose tissue regulator of glucose and fatty acid levels [92]. However, and as mentioned before, *ADIPOQ* expression values were found numerically higher (q = 0.297) in BI with RNA-seq as well as with real-time PCR (*p* = 0.11). AMPK has a key role in the cellular maintenance of ATP levels and is responsible for the regulation of growth and transcriptional controlling programs [93]. In a recent in vivo study in mice [94], AMPK regulation lipolytic activity in adipose tissue was confirmed, due to the direct phosphorylation of hormone sensitive lipase and adipose triglyceride lipase. AMPK as also been found to suppress lipogenesis by phosphorylating SREBP-1 c, inhibiting its transcriptional activity [95]. More recently, purebred IB foetuses have also been found associated with a downregulation of AMPK when compared to crossbred Iberian × Large White pigs in muscle [96]. Observed leptin overexpression would suggest an activated AMPK in AL, however an impaired leptin signalling as proposed by García-Contreras may also be occurring. IGF1 is the first cell response to GH stimulation. This anabolic factor contributes to cell differentiation and homeostasis though is not mandatory in adipose tissue development [97]. IGF1 is essential in the physical stress response that induce myocyte growth and hypertrophy [98], while regulating adipose tissue metabolism by inhibiting lipolysis similarly to insulin [99]. 

Another interesting canonical pathway found significantly affected by the DE genes (*p* < 0.01) include the complex TR/RXR of the nuclear retinoid X family receptors. This pathway is related to thyroid hormone activation, affecting key biological mechanisms such as differentiation, growth and development, lipid and carbohydrate metabolisms, thermogenesis and central nervous system functions [100]. In rats, thyroid hormone has been found to induce differentiation of preadipocytes in white adipose tissue, as well as to increase lipid content in cells, stimulating a variety of lipogenic enzymes including ACLY, ME1 and FASN, etc. [100]. However, in this canonical pathway, the software was not able to predict the direction of the activity pattern.

Other canonical pathways of interest found where no directional activity pattern was predicted included IL-6 and IL-9 signalling, the pentose phosphate pathway, Acetyl-CoA Biosynthesis III (from Citrate), Palmitate Biosynthesis I, Fatty Acid Biosynthesis Initiation II and Stearate Biosynthesis I. IL-6 is a pro-inflammatory cytokine, directly involved in increased fatty acid oxidation and decreased insulin resistance [101]. A group of interleukins, where IL-6 is included, can stimulate AMPK through the activation of signal transducer and activator of transcription 3 (STAT3) which was found upregulated in BI (log2 FC = −0.940, q < 0.05). On the other hand, IL-9 is a cytokine with an associated anti-inflammatory activity [102].

The main canonical pathway associated to the AL breed was Insulin receptor signalling (z-score = 1.633, *p* < 0.05). As mentioned before, insulin sensitivity plays an elemental role in modulating glucose and lipid deposition. Insulin accumulation induce triglyceride deposition while inhibiting lipolysis culminating in more fat mass, where inflammatory cytokines tend to accumulate and promote more insulin resistance in the individuals.

Identification of potential upstream regulators and their effects at a transcriptional level to help explain the obtained DEG’s was accomplished using IPA. A total of 643 regulator molecules were identified (*p* < 0.05, Supplementary Table S5) with 16 predicted to be active in AL (z-score ≥ 2) and 20 predicted to be active in BI (z-score ≤ −2).

Activated upstream regulators found in AL include mainly molecules associated with lipid homeostasis, adipogenesis and/or insulin sensitivity such as ADIPOQ, NR1D1, PPARG, SREBF2, SCAP and MED1. *ADIPOQ* is a gene exclusively expressed in the adipose tissue and its coding protein, adiponectin, is found circulating mostly within the plasma, where it participates in the regulation of several metabolic and hormonal mechanisms [103,104]. Adiponectin is known to trigger AMPK phosphorylation and increase the peroxisome proliferator-activated receptor alpha (PPARA) activity leading to β-oxidation, while being associated with reduced insulin resistance and anti-inflammatory activity through AdipoR1 and AdipoR2 signalling [104]. ADIPOQ circulating levels are inversely related with body fat mass and are higher when a low caloric intake is occurring [104]. In our trial, although animals from both breeds were slaughtered at ~150 kg of body weight, AL pigs have shown a higher ability to deposit fat mass, while BI developed more muscle mass [33], which suggest a higher expression of *ADIPOQ* in BI than in AL. On the other hand, our results indicate that ADIPOQ gene expression is not statistically different between breeds which would suggest that ADIPOQ activation as regulator could be a consequence of differential regulation at other levels, such as post-transcriptional regulation. NR1D1 has been previously described as a key factor in the coordination of the mammalian circadian rhythm with metabolic pathways in several tissues, including adipose tissue [105]. This transcription factor can stimulate adipogenesis and preadipocyte differentiation via a PPAR response element that induces increased expression of various adipogenic markers including *PPARG*, at a later adipogenesis stage [106,107].

The family of sterol regulatory element-binding proteins (SREBFs) is responsible for managing cellular lipid homeostasis in vertebrates by adjusting sterol-regulated lipogenic genes to the cell current needs [108]. SREBF2 is a transcriptional activator homolog to SREBF1 but, unlike the latter, is bounded to stimulate genes related to cholesterol synthesis such as *HMGCR*, *HMGCS*, *MVK* and *LDLR* and not fatty acid synthesis [108,109]. *SCAP* encodes for the protein responsible for the exportation of SREBF’s to the Golgi complex for cholesterol synthesis by binding to the specific SREBF in the endoplasmic reticulum [110].

MED1 is a transcription factor of the Mediator complex and operates by mediating RNA polymerase II-dependent genes. Metabolically, MED1 is essential for adipocyte differentiation and adipogenesis by stimulating *PPARG* expression [111]. Adipogenesis comprehend two main steps. Firstly, early proadipogenic factors such as CEBPB, CEBPD and KLF’s are stimulated and participate in the regulation of enhanceosomes formation which prompt the maturation of differentiated adipocytes by the activation of PPARG and CEBPA [112,113]. In this process, PPARG is recognized to have a master regulatory influence since the other factors cannot stimulate adipocyte differentiation without PPARG [112,114]. Upstream regulators associated to AL confirm the suggested hypothesis of an increased adipogenesis and cholesterol synthesis and uptake in this breed which agrees with the phenotypic data that characterises the fatter AL and the leaner BI genotypes [33,115].

Activated upstream regulators found in BI include molecules involved in the immunoregulatory and inflammatory processes such as MYD88, CCL2, IRF2, RELA and TNF, as well as regulators involved in lipid metabolism and insulin signalling such as INSIG1, INSIG2 and FOXO1. INSIG1 and INSIG2 are identical endoplasmic reticulum proteins and are responsible for mediating feedback control of sterol synthesis by binding to the SCAP protein transporter, preventing activated SREBPs transference to the Golgi apparatus to stimulate lipogenesis [116]. Furthermore, INSIG molecules can also bind to HMGCR, inhibiting its use of cytosolic acetyl-CoA for cholesterol biosynthesis [117]. FOXO1 is regularly expressed in insulin responsive cells such as the adipocytes, is involved in their differentiation and can inhibit lipid synthesis by suppressing the transcriptional activity of PPARG [118]. This protein is also a suggested transcription factor with positive effect in myogenic growth, though studies are not consistent [119]. Other regulators also found to be associated with growth, myogenesis and muscle cell differentiation and proliferation were MEF2C and PDGFB.

Regulator effect analysis identified proliferation of fibroblast cell lines and activation of myeloid cells to be amongst the main functions associated with our determined upstream regulators. This is indicative of connective tissue development and an active state of the innate immune system, probably due to the obesity-associated inflammation, respectively. Additionally, regulator effect analysis together with some of the activated functions in BI may suggest a predominance of the stromal-vascular fraction in the cell population and not adipocytes. The complete list of master regulators and their effects on the regulators and genes in our dataset is shown in Table 2.

**Figure 2:**
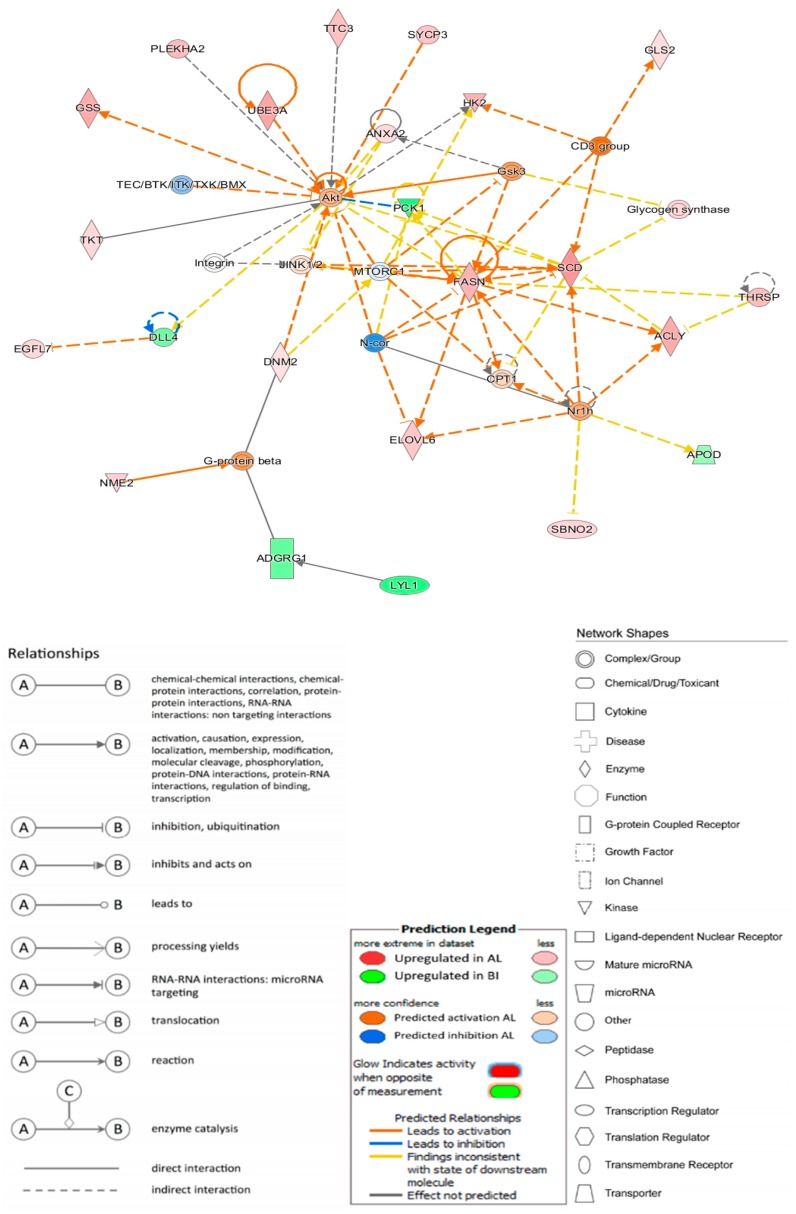
Carbohydrate metabolism, Lipid metabolism and Molecular transport Ingenuity Pathway Analysis (IPA) Network.

Regulators and DE genes were used to conceive causal networks using IPA. One of the main networks established is related to lipid metabolism, carbohydrate metabolism and molecular transport (Figure 2). This network attained a total score of 31, summarizes the associated activity of 23 molecules and emphasizes the central role of *FASN* in the metabolic regulation in adipose tissue.

In normal cells, *de novo* lipid synthesis is only stimulated in case of lipid depletion, since these needs are usually met via absorption of dietary lipids. AL pigs are characterized by a genetic predisposition to be precociously adipogenic which, according to our data, may be associated to a deregulated *de novo* fatty acid synthesis leading to increased fatty acid accumulation, regardless of dietary lipid absorption.

In conclusion, the transcriptome analysis of the Alentejano and Bísaro Portuguese local pig breeds allowed the identification of a total of 458 differentially expressed genes and contributed with valuable insight to what is metabolically occurring in the adipose tissue of these breeds. Our data suggest that the determining factor that differentiate the higher fat deposition in AL is related to an increased *de novo* fatty acid synthesis, due to the upregulation of key genes such as *ACLY*, *FASN* and *ME1*. Cholesterol synthesis is also suggested to be higher in AL via SREBF2, SCAP and PPARG. Lipolytic activity may be more active in BI than AL through GH and AMPK signalling. Moreover, the predicted activation of INSIG1 and INSIG2 in BI suggests that this breed is more sensitive to insulin whereas the AL is less sensitive like the Iberian breed. Our data also suggest that AL pigs, such as previously described in Iberian pigs, have developed patterns of insulin and leptin resistance and obesity-induced chronic inflammation. Moreover, signalling of CD40 may play a dictating role in the development of insulin resistance in AL pigs.

## Figures and Tables

**Table 1:** Gene expression comparison through RNA-seq and Real Time qPCR of the selected genes.

Genes	RNA-seq		Real Time qPCR		Correlation	
	Log2 FC	q-Value	Log2 FC	*p*-Value	*r*	*p*-Value
*ACACA*	0.854	0.116	1.055	0.077	0.986	7.07 × 10^−6^
*ACLY*	1.853	0.005	1.601	0.068	0.864	0.006
*ADIPOQ*	−0.625	0.297	−0.685	0.110	0.707	0.050
*ELOVL6*	1.236	0.009	0.671	0.136	0.650	0.081
*FASN*	1.691	0.002	1.359	0.100	0.920	0.001
*LEP*	1.376	0.037	0.929	0.046	0.739	0.036
*ME1*	2.090	8.42 × 10^−6^	1.008	0.106	0.757	0.030
*PCK1*	−1.959	0.037	−2.660	0.001	0.820	0.013
*SCD*	2.311	0.026	1.351	0.087	0.643	0.086
*n* = 4						

**Table 2:** Causal regulator effects predicted to be activated and inhibited in AL.

Master Regulator	Participating Regulators	Predicted Activation (AL)	Z-Score	*p*-Value	Target Molecules in Dataset
INSIG2	INSIG2	Inhibited	−2.236	3.74 × 10^−6^	5
FLCN	Esrra, FLCN, MTORC1, PPARGC1A	Inhibited	−2.236	7.72 × 10^−6^	20
PKIA	CREB1, Pka, PKIA	Inhibited	−2.887	1.26 × 10^−5^	12
LRAT	Akt, INSR, JAK2, LRAT, RPE65, STAT5a/b	Inhibited	−3.300	3.03 × 10^−5^	18
NRG4	NRG4	Inhibited	−2.000	3.76 × 10^−5^	4
PDGF BB	PDGF BB	Inhibited	−2.333	1.54 × 10^−4^	9
CREB1	CREB1	Inhibited	−2.828	1.69 × 10^−4^	8
GNAS	GNAS	Inhibited	−2.236	1.97 × 10^−4^	5
MFSD2A	MFSD2A	Inhibited	−2.000	4.85 × 10^−4^	4
MEF2C	MEF2C	Inhibited	−2.236	1.47 × 10^−3^	5
HSP27	Hsp27	Inhibited	−2.000	1.63 × 10^−3^	4
NDFIP1	Akt, Jnk, Map3k7, NDFIP1, PTEN, SRC	Activated	2.449	2.31 × 10^−6^	24
NR1D1	NR1D1	Activated	2.236	5.36 × 10^−6^	5
NSUN3	NSUN3	Activated	2.000	5.61 × 10^−6^	4
ALKBH1	ALKBH1	Activated	2.000	5.61 × 10^−6^	4
UCHL3	Akt, AMPK, FOXO1, GSK3B, INSR, UCHL3	Activated	2.449	1.05 × 10^−5^	24
MIR-29B-3P (AND OTHER MIRNAS W/SEED AGCACCA)	Akt, ERK, miR-29b-3p (and other miRNAs w/seed AGCACCA), PMP22	Activated	2.500	1.06 × 10^−5^	16
ARNTL	Akt, ARNTL, CLOCK, NFE2L2	Activated	2.200	2.85 × 10^−5^	25
IDH1	IDH1	Activated	2.236	2.97 × 10^−5^	5
EPCAM	EPCAM	Activated	2.000	3.76 × 10^−5^	4
ATP7B	Akt, ATP7B	Activated	2.887	4.58 × 10^−5^	12
DAP3	DAP3	Activated	2.000	5.36 × 10^−5^	4
BACE1	BACE1, CREB1, Pka	Activated	2.714	7.70 × 10^−5^	11
ATP7B	ATP7B	Activated	2.236	3.98 × 10^−4^	5
MLXIPL	MLXIPL	Activated	2.000	4.02 × 10^−4^	4
SCAP	SCAP	Activated	2.449	4.21 × 10^−4^	6
SREBF2	SREBF2	Activated	2.449	6.09 × 10^−4^	6
GHRL	GHRL	Activated	2.000	8.06 × 10^−4^	4
MED1	MED1	Activated	2.449	1.00 × 10^−3^	6

## Supplementary Materials

The following are available online at https://www.mdpi.com/2073-4425/11/4/422/s1, Table S1: Primer design for qPCR, Table S2: List of differentially expressed genes, Table S3: Enriched biological functions in the set of differentially expressed genes, Table S4: Enriched canonical pathways in the set of differentially expressed genes, Table S5: Predicted upstream regulators for the set of differentially expressed genes.
