# Supplementary material for: Comparative Transcriptomic Analysis of Subcutaneous Adipose Tissue from Local Pig Breeds
Source: Genes (Basel). 2020 Apr 15;11(4):422. doi: 10.3390/genes11040422 (PMC7231169; doi:10.3390/genes11040422)
Supplement: Supplementary file 1 [file genes-11-00422-s001.zip › Supplementary Table 1_Primer design for qPCR.docx]

Supplementary table 1. Primer design for qPCR

| Gene | Sequence (5’-3’) | Product size (bp) | Annealing Temperature (°C) | Accession number | Reference |
| --- | --- | --- | --- | --- | --- |
| *ACTB* | FW: TCGCACTTCATGATCGAGTTG | 138 | 60 | AY550069 | Guo*, et al.* [1] |
|  | RV: CGACGGCCAGGTCATCAC |  |  |  |  |
| *RPL19* | FW: GCTTGCCTCCAGTGTCC | 82 | 58 | AF435591 | Guo, Tang, Wang, Liu and Wang [1] |
|  | RV: GTTGGCGTTGGCGATTT |  |  |  |  |
| *TOP2B* | FW: AACTGGATGATGCTAATGATGCT | 137 | 58 | NM_001258386.1 | Gu*, et al.* [2] |
|  | RV: TGGAAAAACTCCGTATCTGTCTC |  |  |  |  |
| *ACACA* | FW: TCCCAGTGCAAGCAGTATG | 211 | 60 | EF 618729 | Tan*, et al.* [3] |
|  | RV: TGCCAATCCACACGAAGAC |  |  |  |  |
| *ACLY* | FW: GAGGCAGCATCGCAAACTTCAC | 170 | 55 | NM_001105302.1 | Gao*, et al.* [4] |
|  | RV: GGTCTTCCCAACTTCTCCCATC |  |  |  |  |
| *ADIPOQ* | FW: CGTTCAGCATTCAGTGTGG | 178 | 55 | NM214370 | Weber*, et al.* [5] |
|  | RV: TCATTCAATGTTGTGGTAGAGA |  |  |  |  |
| *ELOVL6* | FW: AGAACACGTAGCGACTCCGAAGAT | 177 | 60 | XM_013978957.1 | Benítez*, et al.* [6] |
|  | RV: GACATGCCGACCGCCAAAGATAA |  |  |  |  |
| *FASN* | FW: GCAGGCGCGTGATGGGAATGGTG | 206 | 58 | NM_001099930 | Benítez, Fernández, Isabel, Núñez, Mercado, Gómez-Izquierdo, García-Casco, López-Bote and Óvilo [6] |
|  | RV: GCCCGAGCCCGAGTGGATGAGCA |  |  |  |  |
| *LEP* | FW: GGCCCTATCTGTCCTACGTTGAAG | 237 | 60 | NM_213840.1 | Benítez, Fernández, Isabel, Núñez, Mercado, Gómez-Izquierdo, García-Casco, López-Bote and Óvilo [6] |
|  | RV: TGGAAGGCAGACTGGTGAGGAT |  |  |  |  |
| *ME1* | FW: GCCGGCTTTATCCTCCTCT | 223 | 55 | XM_001924333.5 | [6] |
|  | RV: TCAAGTTTGGTCTGTATTTTCTGG |  |  |  |  |
| *PCK1* | FW: ATGCCTCCTCAGCTCTCAAA | 198 | 60 | NM_001123158.1 | - |
|  | RV: GATGACACCCTCTTCCTCCA |  |  |  |  |
| *SCD* | FW: CCGCCCTGAAATGAAAGATGAC | 184 | 60 | NM_213781.1 | Gao, Zhang, Jiang, Xiao, Wang, Ma, Sun, Li, Deng, Dai, Zhao, Cui, Zhang, Liu and Zhang [4] |
|  | RV: GTAGGCAAACGCCCAGAGCAAG |  |  |  |  |

1. Guo, X.; Tang, R.; Wang, W.; Liu, D.; Wang, K. Effects of dietary protein/carbohydrate ratio on fat deposition and gene expression of peroxisome proliferator activated receptor γ and heart fatty acid-binding protein of finishing pigs. *Livestock Science* **2011**, *140*, 111-116, doi:<http://dx.doi.org/10.1016/j.livsci.2011.02.016>.

2. Gu, Y.R.; Li, M.Z.; Zhang, K.; Chen, L.; Jiang, A.A.; Wang, J.Y.; Li, X.W. Evaluation of endogenous control genes for gene expression studies across multiple tissues and in the specific sets of fat- and muscle-type samples of the pig. *Journal of Animal Breeding and Genetics* **2011**, *128*, 319-325, doi:10.1111/j.1439-0388.2011.00920.x.

3. Tan, B.; Yin, Y.; Liu, Z.; Tang, W.; Xu, H.; Kong, X.; Li, X.; Yao, K.; Gu, W.; Smith, S.B., et al. Dietary L-arginine supplementation differentially regulates expression of lipid-metabolic genes in porcine adipose tissue and skeletal muscle. *The Journal of Nutritional Biochemistry* **2011**, *22*, 441-445, doi:10.1016/j.jnutbio.2010.03.012.

4. Gao, Y.; Zhang, Y.H.; Jiang, H.; Xiao, S.Q.; Wang, S.; Ma, Q.; Sun, G.J.; Li, F.J.; Deng, Q.; Dai, L.S., et al. Detection of differentially expressed genes in the longissimus dorsi of Northeastern Indigenous and Large White pigs. *Genet. Mol. Res.* **2010**, *10*, 779-791.

5. Weber, T.E.; Kerr, B.J.; Spurlock, M.E. Regulation of hepatic peroxisome proliferator-activated receptor alpha expression but not adiponectin by dietary protein in finishing pigs. *Journal of Animal Physiology and Animal Nutrition* **2008**, *92*, 569-577.

6. Benítez, R.; Fernández, A.; Isabel, B.; Núñez, Y.; Mercado, E.D.; Gómez-Izquierdo, E.; García-Casco, J.; López-Bote, C.; Óvilo, C. Modulatory Effects of Breed, Feeding Status, and Diet on Adipogenic, Lipogenic, and Lipolytic Gene Expression in Growing Iberian and Duroc Pigs. *International Journal of Molecular Sciences* **2018**, *19*, 22, doi:10.3390/ijms19010022.
